# Approach to the Dynamic of Carbamazepine and its Main Metabolites in Soil Contamination through the Reuse of Wastewater and Sewage Sludge

**DOI:** 10.3390/molecules25225306

**Published:** 2020-11-13

**Authors:** José Luis Malvar, Juan Luis Santos, Julia Martín, Irene Aparicio, Esteban Alonso

**Affiliations:** Departamento de Química Analítica, Escuela Politécnica Superior, Universidad de Sevilla. C/ Virgen de África, 7, E–41011 Seville, Spain; jmalvar@us.es (J.L.M.); jlsantos@us.es (J.L.S.); jbueno@us.es (J.M.); iaparicio@us.es (I.A.)

**Keywords:** carbamazepine, metabolites, soil, wastewater, sewage sludge, competitive adsorption

## Abstract

The release of pharmaceutically active compounds to the soils through the application of sewage sludge and the irrigation with wastewater, or even with surface water, is constant. The adsorption of these compounds onto the soil is one of the key factors affecting their fate in the environment and their potential environmental risks. In this work, the adsorption of carbamazepine (CBZ) and its metabolites, 3-hydroxy-carbamazepine (3OH-CBZ), carbamazepine-10,11-dihydro-10,11-epoxide (EP-CBZ), and 10,11-dihydro-10-hydroxycarbamazepine (10OH-CBZ), in three Mediterranean soils was evaluated using single-solute and four-solute experiments. The highest adsorptions were measured for 3OH-CBZ, followed by CBZ, EP-CBZ, and 10OH-CBZ, in that order. A high influence of the physicochemical characteristics of the compounds, pH, and soil characteristics in the adsorption of the studied compounds was observed and corroborated by the statistical analysis of the results. Moreover, a good fit was observed in the three isotherm models evaluated (linear, Freundlich, and Langmuir) in single-solute experiments (R^2^ > 0.90). However, a decrease of the measured adsorptions and a worse fit to the isotherm models were observed in the case of multiple-solute experiments. This could be mainly due to the competition established between the studied compounds for the active sites of the soils.

## 1. Introduction

Pharmaceutically active compounds (PhCs) are continuously released into the environment. Their presence in several environmental compartments as wastewater [1,2,3], surface water [1,4] or sewage sludge [5,6,7], and soil [5] has been widely documented. Many of these compounds enter into the soil due to irrigation with treated wastewater [8] or through sewage sludge applied to the agricultural soils as organic amendment [9]. The adsorption of PhCs into the soil is one of the main processes governing not only the fate of these compounds in the terrestrial compartment but also their environmental risks, since it determines their availability for plants and other terrestrial organisms.

This adsorption depends on the physicochemical characteristics of the PhCs (water solubility, pKa, molecular size, etc.), environment (pH, temperature, ionic strength) and soil (texture, organic matter). Recent studies have evaluated the adsorption onto the soil of PhCs as atenolol [10], carbamazepine [10,11], diclofenac [11,12], trimethoprim [10] or sulfamethoxazole [10]. These studies have shown the influence of the physicochemical characteristics of the pharmaceutical [10,11] or environmental conditions [11] in their adsorption behaviour. However, despite the complexity of environmental systems, most of these studies have been carried out using single-solute experiments. Only a few studies have evaluated the potential interactions or competition between PhCs using sorption experiments. Most of these studies were carried out using adsorbent materials such as biochars [13], carbon nanotubes [14] or clay materials [15,16] and, to a less extent, soil [10]. However, there are no studies reporting the potential interaction and competition between PhCs and their metabolites in soils. However, these metabolites are excreted together with the PhCs, in higher quantities than their parent compounds and even can show higher environmental risks [17,18,19,20]. This shows the importance of evaluating the sorption behaviour of these compounds on the soil in multiple-solute systems instead of single-solute systems, as a previous step to the evaluation of this sorption in real systems as wastewater–soil or sludge–soil systems.

Carbamazepine (CBZ) is one of the most prescribed PhCs in the world. It is used to treat epilepsy episodes, bipolar disorder, dysphonia or neuropathic pain [20]. CBZ is mainly excreted by urine (72% of the total of the CBZ consumed), whereas the rest is excreted through feces. The 13% of the CBZ excreted by feces is as parent compounds whereas, in urine, only 1% is excreted unchanged [21]. Until now, more than 30 metabolites have been identified. The main metabolic route comprises the epoxidation of CBZ to carbamazepine-10,11-dihydro-10,11-epoxide (EP-CBZ), followed by conversion to 10,11-dihydro-10-hydroxycarbamazepine (10OH-CBZ) [17]. Additionally, phenolic metabolites such as 1-hydroxy carbamazepine (1-OH CBZ), 2-hydroxy carbamazepine (2-OH CBZ) or 3-hydroxy-carbamazepine (3OH-CBZ) have also been formed in a wide proportion [21]. Only a few works have been reported in the recent literature about the behaviour of CBZ and its metabolites in soil [17,20,22,23]. These studies have been focused on the determination of the adsorption isotherms of the individual compounds. For this purpose, batch experiments are carried out, in most cases, using reference soils. To the best of our knowledge, there are no studies evaluating the adsorption of CBZ and its main metabolites in multiple-solute experiments in which the competition of the compounds for the adsorption onto the soil could occur.

The aims of this work were (I) to evaluate the adsorption of CBZ and its main metabolites, 3OH-CBZ, 10OH-CBZ and EP-CBZ, in three Mediterranean soil using single-solute and four-solute experiments; (II) to assess the influence of physicochemical characteristics of the compounds, environment and soil in their sorption onto the soils; and (III) to evaluate the competition between studied compounds for their adsorption onto the soils.

## 2. Results and Discussion

### 2.1. Preliminary Experiments

Results obtained in batch experiments for the evaluation of the adsorption equilibrium time are presented in Figure S1 (Supplementary Materials). Adsorption values were approximately constant for 24 h for all compounds. Consequently, this time was selected for the subsequent experiments. Regarding the soil/solution ratio (Figure S2), the data obtained for 10OH-CBZ and EP-CBZ did not show significant adsorption variations with the increase of the amount of soil. Only a slight increase of their adsorption was observed. By contrast, CBZ and 3OH-CBZ exhibited a great dependency with the amount of soil used. Their adsorption increases with the increase of the ratio soil/solution. This behaviour could be due to the increase of soil active sites at higher soil amount that was observed in major extension for those compounds showing the higher adsorption. For this reason, the highest ratio (5:10 (*w/v*)) was selected as the optimal ratio.

### 2.2. Adsorption of Studied Compounds

#### 2.2.1. Single-Solute Systems

**Figure 1:**
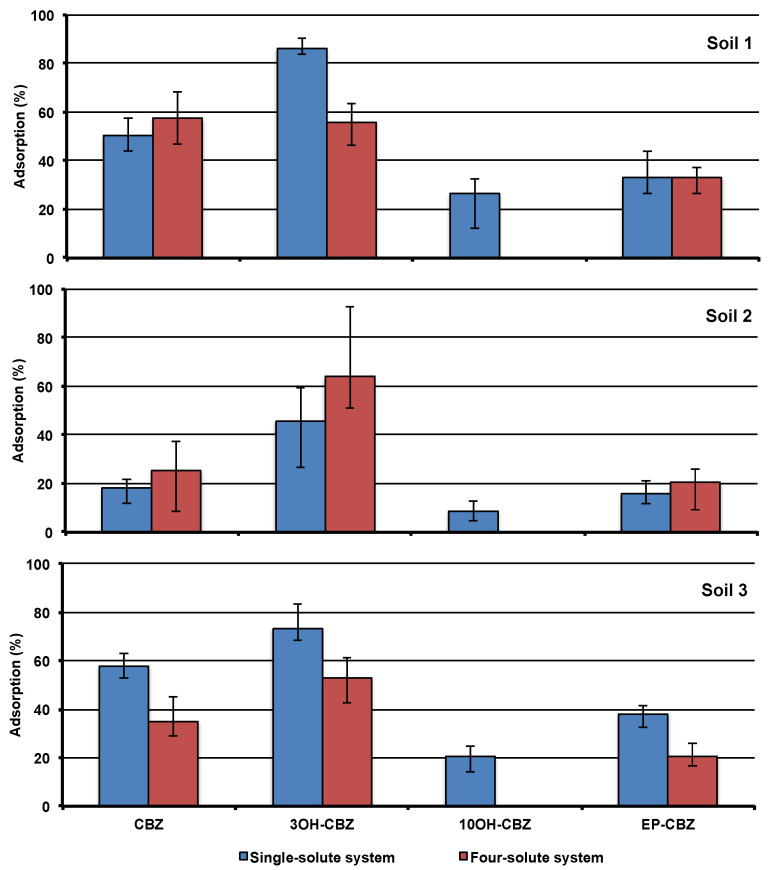
Adsorption of carbamazepine (CBZ) and its main metabolites, 3-hydroxy-carbamazepine (3OH-CBZ), carbamazepine-10,11-dihydro-10,11-epoxide (EP-CBZ), and 10,11-dihydro-10-hydroxycarbamazepine (10OH-CBZ) in the studied soil in single-solute system and in four-solute system.

In all studied soils, the adsorption capacity follows the same order: 3OH-CBZ > CBZ > EP-CBZ > 10OH-CBZ (Figure 1). This distribution was similar to those measured by other authors [17,23] and could be explained considering the molecular structure of studied compounds.

For example, Paz et al. (2016) [23] related the different adsorptions of CBZ, EP-CBZ, and 10,11-dihydro-10,11-dihydroxycarbamazepine (DiOH-CBZ) with their heterogeneous charge distribution density. The electronegative oxygen atoms sited in the epoxide- and hydroxyl groups of the EP-CBZ and 10OH-CBZ, respectively, allow them to form hydrogen bonds with water molecules. These interactions can inhibit their adsorption onto the soil surface [23]. This would explain the low affinity of 10OH-CBZ for the studied soils. The high affinity of 3OH-CBZ could be explained by the position of the hydroxide group, sited near to the amino group. This hydroxide group can contribute to enhanced stability of the molecule on the soil surface due to the formation of hydrogen bonds with the functional groups of the soil organic matter, as it has been described for other compounds [23].

Considering the influence of pH on the adsorption, no significant differences were observed for EP-CBZ in soil 1 (adsorptions measured were from 25 to 32%, standard deviation 2.8%) and soil 3 (from 27 to 33%, standard deviation 2.3%) at different pH values (Figure S3). In the case of CBZ and 10OH-CBZ, a slight decrease of their adsorption was measured as pH grows from 2 to 12. In the case of 3OH-CBZ, a strong influence of pH was observed. Mean adsorption of 3OH-CBZ measured between pH 2 and 10 was 56.0 ± 1.7 and 51.5 ± 1.5% for soils 1 and 3, respectively, while the adsorption measured at pH 12 was 23 ± 3.0% for soil 1 and 6.07 ± 0.14% in the case of soil 3. These results could be explained considering the pK_a_ value of the studied compounds. EP-CBZ (pK_a_ = 16.0) was not affected by the pH between 2 and 12. The compounds CBZ and 10OH-CBZ (pK_a_ 12.8 and 13.9, respectively) are partially deprotonated at pH 12. At this pH value, the adsorption could be inhibited by electrostatic repulsion between the ionic form of the compounds and the soil surface; consequently, the ionic forms showed higher affinity by the liquid phase. This was particularly observed on 3OH-CBZ (pK_a_ = 9.19), which is in its ionic form at pH 12.

Soil 2 showed the lowest adsorption capacity, followed by soil 1 and soil 3, in that order. This could be related to the high amount of sand (74%) and low organic matter (0.58%) of the soil 2, which facilities the permeability and leaching of these pollutants as it was described previously [10,17,23,24]. In the case of soil 1 (24.6% of sand and 0.91% of organic matter), the adsorptions measured were in the ranges 84–90%, 44–58%, 27–44%, and 12–33% for 3OH-CBZ, CBZ, EP-CBZ and 10OH-CBZ, respectively. For soil 3 (65.6% of sand and 2.01% of organic matter), the same order was observed: 3OH-CBZ (68–83%) > CBZ (53–63%) > EP-CBZ (32–42%) > 10OH-CBZ (14–25%). As can be seen, soils 1 and 3 showed similar adsorption capacity of each of the studied compounds, except in the case of 3OH-CBZ, which showed significant differences (Student *t*-test: t_cal_ = 4.727, t_tab_ = 2.228, *p* > 0.05). The influence of soil characteristics in the adsorption of the studied compounds was corroborated by the statistical multifactorial analysis. The correlation matrix obtained in the single-solute experiments is shown in Table S1 (supplementary material). High correlations (absolute value higher than 0.70) were obtained between the adsorptions of the compounds with hydroxide groups (3OH-CBZ and 10OH-CBZ) and the silt and clay content of the soil. Moreover, high correlations were obtained between the adsorptions of compounds without hydroxide groups (CBZ and EP-CBZ) with the organic matter. Considering factor analysis, two factors, with eigenvalues higher than 1 (Factor 1: 5.56; Factor 2: 4.44), were obtained using the data obtained in the single-solute experiments (Table S2). These factors could indicate different adsorption mechanics of the compounds depending on their functional groups and the influence of the physicochemical characteristics of the soil on these mechanics. The first factor, accounting for 56% of the total variability, was composed of 3OH-CBZ and 10OH-CBZ and the texture (sand, silt, and clay content) of the soil. The second factor, accounting for 44% of the total variability, was composed of CBZ and EP-CBZ and the organic matter contents. This indicates a higher interaction of polar compounds (3OH-CBZ and 10OH-CBZ) with silt and clay and a higher interaction of non-polar compound (CBZ and EP-CBZ) with organic matter of the soil.

#### 2.2.2. Four-Solute Systems

Significant differences were not observed between adsorptions measured in soil 2 for the four-solute systems (Figure 1) with those measured in the single-solute systems. In the case of soil 1, a significant reduction of the adsorption was measured for 3OH-CBZ (Student t-test: t_cal_ = 12.0, t_tab_ = 2.28, *p* < 0.05) and 10OH-CBZ (from 26.4% in the case of single-solute experiments to no adsorption in the case of four solute experiments). Considering soil 3, all studied compounds showed lower adsorptions in the four-solute experiments (Student *t*-test CBZ: t_cal_ = 5.167, t_tab_ = 2.571, *p* > 0.05; 3OH-CBZ: t_cal_=5.670, t_tab_=2.228, *p* < 0.05; EP-CBZ: t_cal_=7.825, t_tab_=2.228, *p* < 0.05). These results could be due to the competition of the studied compounds for their adsorption onto the soils in the four-solute system. This was especially important in the case of soil 3. The multivariable analysis showed relations between the adsorption of CBZ and its metabolites and the texture of the soils (Table S1). Positive correlations were obtained between the adsorptions and silt and clay, and negatives with coarse sand. Moreover, in contrast with the single-solute experiments, correlations between adsorptions and organic matter were not observed. These results indicate a low influence of the organic matter in the adsorption of CBZ and its metabolites, which is contrary to the results obtained in the single-solute experiments. The same results were obtained in the factorial analysis. Two factors, with eigenvalues higher than 1 (Factor 1: 6.05; Factor 2: 2.95), were obtained (Table S3). The first factor, accounting for 67% of the total variability, was composed of the studied compounds and the texture parameters. The second factor, accounting for 33% of the total variability, was composed only of the organic matter. These results show that in multiple-solute systems the silt and clay content of the soil could play a more important role than organic matter on the adsorption of organic pollutants onto the soil.

### 2.3. Adsorption Isotherms

#### 2.3.1. Single-Solute Systems

**Figure 2:**
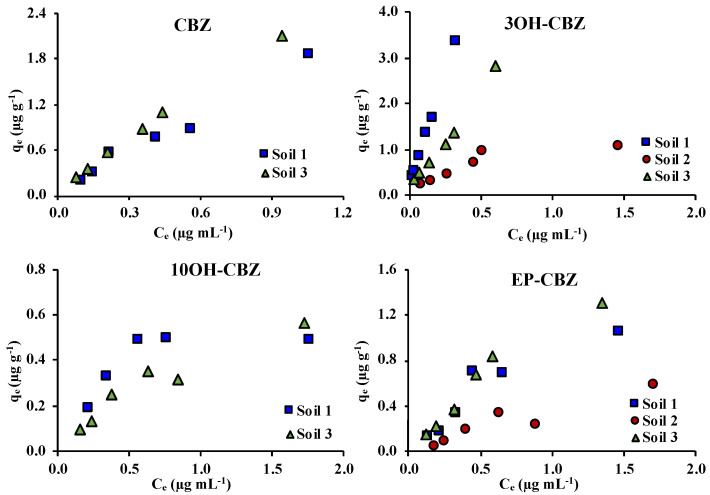
Adsorption curves for CBZ and its metabolites in studied soils in the single-solute systems; q_e_: amount of compound adsorbed; C_e_: concentration of compound in the equilibrium solution.

Adsorption curves of CBZ and its metabolites in the single-solute systems are shown in Figure 2. In all cases, an increase of the adsorbed amount was observed with the increase of the initial concentration. Moreover, graphically, the saturation of the soils was not observed, except in the case of 10OH-CBZ in soil 1. This could be due to the low concentration range evaluated in this work. Similar results have been obtained in other studies carried out at concentrations up to 10 µg mL^−1^ [10,17,20].

Linear, Freundlich and Langmuir isotherms obtained in the single-solute experiments are shown in Figures S4–S13 (Supplementary Materials). Table 1 shows the parameters calculated for each of the studied adsorption models. In general, good fitting (R^2^ > 0.90) was obtained in the most cases for all evaluated models. EP-CBZ showed R^2^ values slightly lower than 0.9. Low correlation coefficients were obtained only in the case of 10OH-CBZ in soil 1 for linear (0.4142) and Freundlich (0.6911) models. The good fitting obtained, especially in the case of the linear model, has been described as indicative of the interaction of hydrophobic compounds and the soil organic matter [25]. This could corroborate the influence of organic matter on the adsorption of the studied compounds onto the soil.

Considering the water/soil partition coefficient (K_d_), the lineal correlation obtained could imply a constant availability of the active sites at the evaluated concentrations [26]. The obtained K_d_ values were similar to those previously reported [17,20,23]. The highest K_d_ values were obtained for 3OH-CBZ (9.89, 1.58 and 4.29 in soils 1, 2 and 3, respectively) and CBZ (1.65 and 2.12 in soils 1 and 3, respectively). EP-CBZ (0.68, 0.32 and 0.93 in soils 1, 2 and 3) and 10OH-CBZ (0.14 and 0.28 in soils 1 and 3) showed the lowest K_d_ values. The differences between the K_d_ values of the studied soils show the influence of the physicochemical characteristics of the soils, as it was described previously [20]. These K_d_ values show a high mobility of the compounds in the studied soils [27], especially in soil 2. Moreover, negative correlations were observed between K_d_ and pK_a_ values in the case of soils 1 and 3 (Pearson correlation coefficient of −0.87 and −0.76 were obtained for soils 1 and 3, respectively). This shows the important influence of the pH in the adsorption of some of the studied compounds onto the soils as it was described above.

Considering the Freundlich model, the parameter 1/n was lower than 1 in all cases, except for EP-CBZ (Table 1). These values indicate a favourable adsorption process [28]. Moreover, 1/n values were close to 1 in all cases (from 0.709 to 1.005), except for 10OH-CBZ in soil 1. This explains the high correlation coefficients obtained in both linear and Freundlich models since when 1/n is equal to 1, the Freundlich equation becomes linear. As a result, K_d_ and K_F_ obtained were similar.

Considering Langmuir model, a good fit was measured for all compounds and soils. However, the highest concentrations applied in this work were not high enough to achieve the soil saturation and, consequently, to evaluate the suitability of this model. Indeed, non-realistic values for q_max_ and k_L_ were obtained in the case of EP-CBZ (Table 1).

#### 2.3.2. Four-Solute Systems

Adsorption curves of the studied compounds in the soils in the four-solute systems are shown in Figure 3. Adsorption isotherms obtained in four-solute experiments are shown in Figures S4–S13. The parameters calculated for each of the studied adsorption models are shown in Table 1.

A good fitting was obtained for all evaluated isotherms (R^2^ values higher than 0.9100, 0.8947 and 0.8535 for Linear, Freundlich, and Langmuir isotherms models), except in the case of soil 2 for which low correlation coefficients were obtained in the case of Freundlich and Langmuir isotherms of 3OH-CBZ (0.7464 and 0.7501, respectively) and for linear and Freundlich isotherms (0.4900 and 0.8079, respectively) for EP-CBZ. CBZ was not adsorbed in soil 2 and 10OH-CBZ was not adsorbed in any of the studied soils.

For most of the studied compounds a decrease of the K_d_ and K_F_ values was observed, except for CBZ and EP-CBZ in soil 1, which indicates a lower adsorption than those measured in the single-solute experiments. This decrease of the K_d_ and K_F_ values was especially important in the case of 3OH-CBZ. For example, in soil 1, K_d_ and K_F_ values were from 9.89 and 7.12, respectively, to 1.53 and 1.71. Moreover, 1/n values obtained in four-solute systems were lower than those obtained in single-solute systems. This could indicate the saturation of adsorption sites available to the studied compounds, which results in a lower adsorption. These results could be due to a competition among the studied compounds for active sites of the soils.

## 3. Materials and Methods

### 3.1. Chemicals and Reagents

HPLC-grade methanol (MeOH) and water were supplied by Romil (Barcelona, Spain). Analytical-grade formic acid (98%) and calcium chloride anhydrous (CaCl_2_) were obtained from Panreac (Barcelona, Spain). CBZ and its metabolites 3OH-CBZ, 10OH-CBZ and EP-CBZ were purchased from Sigma-Aldrich (St. Louis MO, USA). The structural formula and physicochemical properties of these compounds are shown in Table 2. Individual stock standard solutions were prepared at 1000 µg·mL^−1^ in MeOH and stored at −18 °C. Working solutions of 100 µg·mL^−1^ were prepared using 0.01 M CaCl_2_ aqueous solution in order to avoid the co-solvent effect due to a high amount of methanol in the adsorption experiments. Other solutions where prepared by dilution of working solution using 0.01 M CaCl_2_ aqueous solution and stored in dark at −18 °C.

### 3.2. Sampling and Soil Preparation

Mediterranean soils with different physicochemical characteristics and widely distributed in Mediterranean region were selected. Soil 1 was an alluvial nature soil sited in the plain of the main rivers from Europe; soils 2 and 3 were terra rossa and cambisol soils and are widely extended in several countries from Europe such as Spain, Italy, Greek, France or Germany.

The three agricultural surface soils (0–20 cm) were collected from Seville (SW Spain). Soils were freeze-dried in a Cryodos-50 lyophilizer (Telstar, Terrasa, Spain), homogenized, sieved (particle size < 2 mm) and kept in glass bottles and stored at −18 °C.

The characterization of the soils was carried out by the determination of texture (fine sand (0.2–0.02 mm), coarse sand (2–0.2 mm), silt (0.02–0.002) and clay (less than 0.002)), pH and electrical conductivity of a 1:2.5 (*w/v*) soil:water suspension, and organic matter (Walkley–back method). The physicochemical characteristics of the three studied soils are shown in Table 3. The presence of CBZ and its metabolites in the selected soil were investigated by the analysis of the soils according to Malvar et al. (2020) [34]. Concentrations of studied compounds were lower than detection limits in all studied soils.

### 3.3. Batch Experiments

#### 3.3.1. Preliminary Experiments

Adsorption experiments were carried out in triplicate following the OECD guideline 106 [35]. Conditions applied in the all batch experiments are summarized in Table S4 (Supplementary Data). Preliminary tests were carried out in order to obtain the appropriate conditions for the adsorption experiments. Two parameters were evaluated in the preliminary experiments: equilibrium time and soil/solution ratio. The studies reported in the literature about the adsorption of CBZ and related compounds onto the soil were carried out applying an equilibrium time between 18 to 24 h [10,17,27]. In order to obtain the equilibrium time, in this work, this parameter was evaluated in one of the studied soils from 10 min to 24 h. Soil 1, which showed an adsorption capacity between the other studied soils in previous experiment (data no showed), was selected. A soil/solution ratio of 2:10 (w:v) was applied and a concentration of target compounds of 1 µg mL^−1^ was selected according to adsorption experiment reported in the literature [10,20,36]. All experiments were carried out at room temperature (25 ± 2 °C). For each compound, 2 g of soil were placed into a 50 mL falcon centrifuge tube and 9 mL of 0.01 M CaCl_2_ solution was added. The mixture was shaken at 40 rpm in a rotator shaker (LLG-uniLOOPMIX2) for 24 h to pre-equilibrate the soil. Then, 1 mL of 0.01 M CaCl_2_ aqueous solution containing 10 µg·mL^−1^ of each tested compound was added. The tubes were agitated at 40 rpm for 10, 20, 30, 40, 50 min and 1, 2, 12, and 24 h. Blank experiments (without soil) were carried out (for 24 h) in order to evaluate the potential adsorption of studied compounds into the plastic walls of the falcon tubes and their degradation during batch experiments. All experiments were carried out by triplicate. After agitation, the tubes were centrifuged at 4000 rpm for 10 min. The supernatant was filtered through 0.22 µm nylon filter and stored at −18 °C until analysis.

The optimal soil/solution ratio was determined for the three studied soils. The appropriate amounts of soil (1, 2, 4, and 5 g), corresponding to 1:10, 2:10, 4:10, and 5:10 soil/solution ratio (*w/v*), were placed in a falcon centrifuge tube and 9 mL of 0.01 M CaCl_2_ solution were added. Ratio values higher than 5:10 (*w/v*) were not tested in order to avoid the saturation of aqueous solution. Batch experiments were carried out (by triplicate) according to what was described above, including blank experiments and pre-equilibration. Centrifuge tubes were shacked for 24 h at 40 rpm and then, centrifuged at 4000 rpm for 10 min. The supernatant was filtered at 0.22 µm nylon filter and stored at −18 °C until analysis.

#### 3.3.2. Influence of pH Experiments on the Adsorption of Studied Compounds

The evaluation of the influence of pH on the adsorption of studied compounds onto the soil was evaluated at different pH values (pH 2, 4, 6, 8, 10, and 12) in two of the studied soils (soils 1 and 3). These soils were those where the highest adsorptions were measured in a previous experiments (data no showed). These tests were carried out by triplicate. Batch experiments were done as described in the preliminary experiments section including soil pre-equilibration, blank experiments and shaking for 24 h at 40 rpm. The pH of the aqueous phase was adjusted using 1 M aqueous solutions of HCl or 1 M aqueous solution of NaOH.

#### 3.3.3. Adsorption Isotherm Experiments

For adsorption isotherm experiments, a soil/solution ratio of 5:10 (*w:v*) and an equilibrium time of 24 h was selected. The experiments were carried out in triplicate at room temperature (25 ± 2 °C) for the three studied soils. In addition, control samples (falcon tubes without soil) were processed to evaluate the adsorption of the compounds on the vessels.

Aliquots of 5 g of soil were placed into a plastic tube and 9 mL of 0.01 M CaCl_2_ were added. The tubes were maintained under agitation during 24 h at 40 rpm to equilibrate the soils. Six different concentrations were used to obtain adsorption isotherms. Each concentration was obtained by the addition of 1 mL of a working solution containing the proper concentration of the studied compounds. The studied concentrations were 0.2, 0.3, 0.5, 0.8, 1, and 2 mg·L^−1^ (approximately from 0.8 to 8.5 µmol·L^−1^ for each compound). These concentrations were higher than those usually measured in the environment for these compounds. However, these concentrations were selected considering the concentration ranges used in similar studies reported in the literature for CBZ and its metabolites [20] and for others organic pollutants [27,37]. Moreover, these concentrations were necessary to obtain statistically robust results difficult to obtain at usual environment concentrations. After that, the tubes were agitated for 24 h at 40 rpm, centrifuged for 10 min at 4000 rpm and filtered through a 0.22 µm syringe filter. The described process was applied separately for each target compound (in single-solute experiment) and simultaneously with a mixture of all compounds at the same concentrations (in four-solute experiment).

### 3.4. Chromatographic Determination

Supernatant obtained from preliminary and adsorption isotherms experiments were analyzed by direct injection on LC-MS/MS system according to Malvar et al., 2019 [19]. The equipment used was an Agilent 1200 series HPLC (Agilent, Santa Clara, CA, USA) coupled to a 6410 triple quadrupole (QqQ) mass spectrometer (MS) equipped with an electrospray ionization source (ESI). Chromatographic conditions are summarized in Table S5. MS parameters were as follows: capillary voltage, 4000 V; drying gas flow rate 9 L·min^−1^; drying gas temperature 350 °C; and nebulizer pressure; 40 psi. Instrument control and data acquisition were carried out with MassHunter software (Agilent, USA). The MS/MS parameters for each compound are given in Table S5 (Supplementary Materials).

### 3.5. Method Performance and Quality Control

The analytical method was validated by the determination of inter- and intraday precision and limit for detection and quantification. Precision was determined by the injection by triplicate of a matrix-matched standard solution at three concentration levels. Limit of detection (LOD) and quantification (LOQ) were determined as the concentration corresponding to signal-to-noise ratios of 3 and 10, respectively. LOD and LOQ were determined by the injection of matrix-matched calibration standards at low concentrations. Inter and intraday precision and limit of detection and quantification are shown in Table S6. The determination of the concentrations was carried out using matrix-matched calibration curves [19]. For this purpose, 5.0 g of soil was added to a Falcon tube and 9 mL of 0.01 M CaCl_2_ solution was added. The tube was shaken for 24 h at 40 rpm. Then, Falcon tube was centrifuged (15 min at 4000 rpm) and the supernatant was used for the preparation of the matrix-matched calibration standards.

For each batch of samples, in addition to matrix-matched calibration curves and extracts obtained in batch experiments, quality control standard and blank samples were measured.

### 3.6. Data Analysis

Adsorption percentage of the compound adsorbed onto the soil was calculated following the expression:

Ads (%) = [(C_i_-C_e_)/C_i_] × 100 (1)

where C_i_ is the initial concentration (µg mL^−1^) and C_e_ is the concentration in the equilibrium (µg·mL^−1^).

The three most widely used models of isotherms were evaluated (Linear, Freundlich and Langmuir). The equilibrium concentration (q_e_, µg·g^−1^) of each tested compound adsorbed onto the soil was calculated as the difference between the solution concentrations before and after the experiment, as follow:

q_e_= (C_i_–C_e_) · V/m (2)

where Ci is the initial concentration measured in the solution (µg·mL^−1^), Ce is the concentration measured in the solution in the equilibrium (µg·mL^−1^), V is the solution volume (mL), and m is the soil mass (g).

Linear model Equation (3) is the simplest adsorption model and assumes a proportional adsorption. This model can be written as:

q_e_ = K_d_ × C_e_ (3)

where K_d_ is the solution-soil distribution coefficient (mL g^-1^).

Freundlich and Langmuir models Equations (4) and (5), respectively, are not linear models and can be expressed as follow:

q_e_ = K_F_ × C_e_^1/n^ (4)

1/q_e_ = 1/q_max_ + 1/(K_L_q_max_C_e_) (5)

where K_F_ is the Freundlich coefficient, which is related to the multilayer adsorption capacity ((µg·g^−1^)) (mL·µg^−1^)^1/n^), K_L_ (mL·g^−1^) are Langmuir constants, which are related to the adsorption bonding energy; 1/n is the heterogeneity factor, which indicates the adsorption intensity; and q_max_ (µg·g^−1^) is the maximal adsorption capacity.

All models were fitted using Excel and applying the correlation coefficient (R^2^) to judge the fitting goodness of different models to data.

Statistical techniques, correlation, and factor analysis were used to evaluate the existence of potential correlations between the adsorption of studied compounds and the characteristics of the studied soils. For this purpose, the mean adsorption of each studied compound and the physicochemical characteristics of the soils (Table 3) were used as variables and each of the soils as cases. Statistical analysis was carried out using Statistical 10.0 software for Windows.

## 4. Conclusions

In this work, the adsorption of CBZ and its main metabolites in three Mediterranean soils have been studied in single-solute and four-solute systems. The adsorptions measured in the soils were in the order: 3OH-CBZ > CBZ > EP-CBZ > 10OH-CBZ. This could be explained considering the functional groups of the compounds and their position in the molecules. The adsorption of 3OH-CBZ on the soils was strongly affected by the pH of the aqueous solution. In the case of the other studied compounds, low influence was observed, mainly due to their pK_a_ values.

The influence of physicochemical characteristics of the soils on the adsorption of the target compounds was evaluated in both single-solute and four-solute experiments. Considering single-solute systems, a high correlation was obtained between the adsorption of CBZ and EP-CBZ and the organic matter of the soil. However, in the case of hydroxylated compounds (3OH-CBZ and 10OH-CBZ), the high correlation was measured between their adsorptions and the texture of the soils. Moreover, a good fit was measured in all studied adsorption isotherms in the single-solute experiments. These could be related with the low concentration ranges evaluated and the excess of active sites in the studied soils.

A decrease of the adsorption of CBZ and its metabolites was observed in the four-solute experiments. Moreover, in spite of the good fit observed in the isotherms evaluated, this fit was, in most of the cases, lower than those observed in the single-solute experiments. Moreover, the study of the adsorption isotherms showed the saturation of adsorption sites available to the studied compounds. These results could be due to the competition between the studied compounds, and a higher influence of the texture of the soil was observed.

The results obtained in this work show the importance of evaluating adsorption behaviour of the studied compounds in the complex real environments as soils amended with compost or sludge from wastewater treatment plants or even irrigated with treated wastewater.

## Figures and Tables

**Figure 3:**
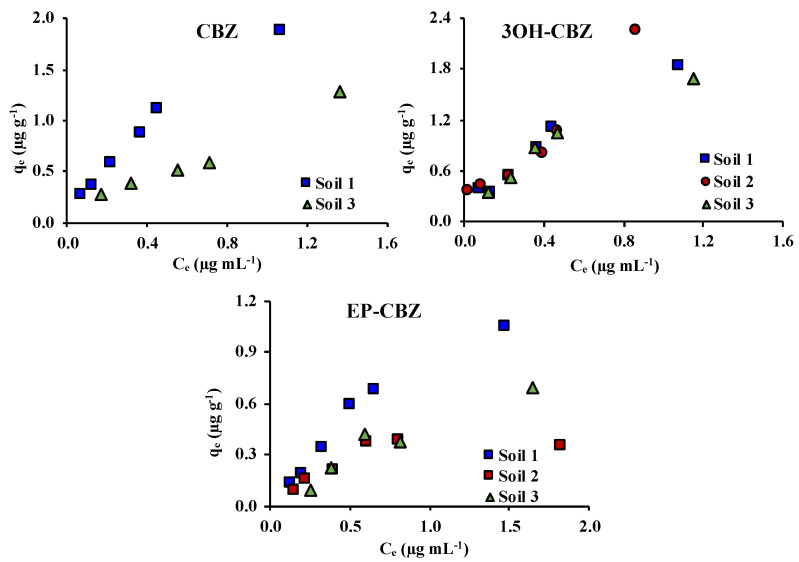
Adsorption curves for CBZ and its metabolites in studied soils in the four-solute systems; q_e_: amount of compound adsorbed; C_e_: concentration of compound in the equilibrium solution.

**Table 1:** Soil/water partition coefficient (K_d_), Freundlich constant (K_F_), Freundlich exponent (1/n), Langmuir constant (K_L_), and maximal sorption capacity (q_max_) of CBZ and its metabolites measured in the studied soils.

	Henry		Freundlich			Langmuir		
	K_d_ (mL g^−1^)	R^2^	K_F_ ((µg g^−1^)) (mL µg^−1^)^1/n^)	1/n	R^2^	K_L_ (mL g^−1^)	q_max_ (µg g^−1^)	R^2^
**Single-Solute System**								
CBZ								
Soil 1	1.65	0.9783	1.69	0.848	0.9698	0.45	5.30	0.9801
Soil 2	-	-	-	-	-	-	-	-
Soil 3	2.12	0.9975	2.19	0.842	0.9990	1.05	3.38	0.9914
3OH-CBZ								
Soil 1	9.89	0.9974	7.12	0.762	0.9835	8.27	2.71	0.9473
Soil 2	1.58	0.9906	1.52	0.803	0.9692	2.04	1.54	0.9176
Soil 3	4.29	0.9895	3.31	0.709	0.9713	5.73	1.97	0.9525
10OH-CBZ								
Soil 1	0.14	0.4142	0.49	0.432	0.6911	1.27	0.98	0.9055
Soil 2	-	-	-	-	-	-	-	-
Soil 3	0.28	0.9225	0.41	0.723	0.9501	0.56	1.21	0.9830
EP-CBZ								
Soil 1	0.68	0.8432	0.94	0.953	0.9052	-0.21	-4.35	0.9599
Soil 2	0.32	0.8879	0.37	1.005	0.8923	-0.46	-0.65	0.9249
Soil 3	0.93	0.9353	1.16	0.934	0.9711	0.04	35.06	0.9926
**Four-Solute System**								
CBZ								
Soil 1	1.60	0.9732	1.82	0.713	0.9911	2.57	1.83	0.9584
Soil 2	-	-	-	-	-	-	-	-
Soil 3	0.83	0.9747	0.86	0.702	0.9445	0.83	1.17	0.9287
3OH-CBZ								
Soil 1	1.53	0.9679	1.71	0.646	0.9285	4.43	1.37	0.7284
Soil 2	2.23	0.9405	1.45	0.387	0.7464	55.84	0.84	0.5626
Soil 3	1.25	0.9461	1.64	0.695	0.9736	17.29	2.45	0.9636
10OH-CBZ								
Soil 1	-	-	-	-	-	-	-	-
Soil 2	-	-	-	-	-	-	-	-
Soil 3	-	-	-	-	-	-	-	-
EP-CBZ								
Soil 1	0.68	0.9271	0.90	0.891	0.9680	0.08	12.57	0.9912
Soil 2	0.14	0.4900	0.36	0.559	0.8074	0.84	0.80	0.9601
Soil 3	0.39	0.9100	0.50	0.966	0.8947	−0.52	−1.18	0.9147

**Table 2:** Physical-chemical properties of the target compounds.

Compound	Molecular Weight	pK_a_	Log K_ow_	Water Solubility (mg/L)	Structure
Carbamazepine (CBZ)	236.27	13.9 ^a^	2.5 ^b^	0.15 ^f^	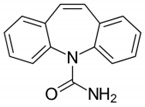
3-Hydroxycarbamazepine (3OH-CBZ)	252.27	9.19 ^c^	2.41^d^	n.a.	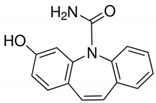
10,11-Dihydro-10-hydroxy carbamazepine (10OH-CBZ)	254.28	12.8 ^d^	0.93 ^e^	n.a.	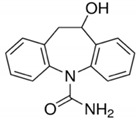
Carbamazepine-10,11-epoxide (EP-CBZ)	252.27	16.0 ^d^	1.0 ^b^	1.34 ^f^	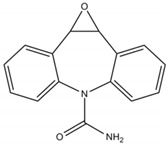

^a^ Rosal et al. 2010 [29]; ^b^ Muñoz et al. 2008 [30]; ^c^ Lee et al. 2011 [31]; ^d^ Huntscha et al. 2012 [32]; ^e^ Miao et al. 2005 [33]; ^f^ Obtained from Human metabolome databases HMDB (http://www.hmdb.ca) (accessed April 2020); n.a.: not available.

**Table 3:** Physical-chemical properties of the studied Mediterranean soils.

	Soil 1	Soil 2	Soil 3
Fine sand, wt.%	16.4	4.70	14.0
Coarse sand, wt.%	8.20	69.5	51.6
Silt, wt.%	44.5	5.80	18.4
Clay, wt.%	30.8	19.9	16.0
pH	8.27	8.21	8.06
EC, µS·cm^−1^	129	75	126
OM, wt.%	0.91	0.58	2.01

EC: Electrical conductivity; OM: organic matter.

## Supplementary Materials

The following are available online. Figure S1. Evaluation of the equilibrium time on the sorption of CBZ, 3OH-CBZ, 10OH-CBZ, and EP-CBZ onto the soil; Figure S2. Evaluation of the soil/solution ratio on the sorption of CBZ, 3OH-CBZ, 10OH-CBZ, and EP-CBZ onto the soil; Figure S3. Evaluation of the influence of pH on the sorption of CBZ, 3OH-CBZ, 10OH-CBZ, and EP-CBZ onto (A) soil 1 and (B) soil 3; Figure S4. Linear, Freundlich and Langmuir models of CBZ adsorption on soil 1 in single-solute and four-solute systems; Figure S5. Linear, Freundlich and Langmuir models of CBZ adsorption on soil 3 in single-solute and four-solute systems; Figure S6. Linear, Freundlich and Langmuir models of 3OH-CBZ adsorption on soil 1 in single-solute and four-solute systems; Figure S7. Linear, Freundlich and Langmuir models of 3OH-CBZ adsorption on soil 2 in single-solute and four-solute systems; Figure S8. Linear, Freundlich and Langmuir models of 3OH-CBZ adsorption on soil 3 in single-solute and four-solute systems; Figure S9. Linear, Freundlich and Langmuir models of 10OH-CBZ adsorption on soil 1 in the single-solute system; Figure S10. Linear, Freundlich and Langmuir models of 10OH-CBZ adsorption on soil 3 in the single-solute system; Figure S11. Linear, Freundlich and Langmuir models of EP-CBZ adsorption on soil 1 in single-solute and four-solute systems; Figure S12. Linear, Freundlich and Langmuir models of EP-CBZ adsorption on soil 2 in single-solute and four-solute systems; Figure S13. Linear, Freundlich and Langmuir models of EP-CBZ adsorption on soil 3 in single-solute and four-solute systems (Table S1). Matrix correlation of CBZ, 3-OH CBZ, 10-OH CBZ and EP-CBZ in single and four solute systems, texture soils, organic matter and organic carbon; Table S2. Results of the factorial analysis considering adsorption of studied compounds in the single-solute experiments and the physicochemical characteristics of the soils as variables and soils as cases; Table S3. Results of the factorial analysis considering adsorption of studied compounds in the four-solute experiments and the physicochemical characteristics of the soils as variables and soils as cases; Table S4. Conditions applied in the batch experiments; Table S5. LC-MS/MS parameters (Table S6). Instrumental limits of detection (LOD), instrumental limits of quantitation (LOQ) and intra- and interday precision, measured as relative standard deviation (n = 3) of the optimized methods.
