# Supplementary material for: Approach to the Dynamic of Carbamazepine and its Main Metabolites in Soil Contamination through the Reuse of Wastewater and Sewage Sludge
Source: Molecules. 2020 Nov 13;25(22):5306. doi: 10.3390/molecules25225306 (PMC7709016; doi:10.3390/molecules25225306)

Approach to the dynamic of carbamazepine and its main metabolites in soil contamination through the reuse of wastewater and sewage sludge

José Luis Malvar, Juan Luis Santos, Julia Martín, Irene Aparicio, Esteban Alonso*

Departamento de Química Analítica, Escuela Politécnica Superior, Universidad de Sevilla. C/ Virgen de África, 7, E–41011 Seville, Spain

* Correspondence: ealonso@us.es; Tel: +34 954 55 62 50

Table captains:

Table S1. Matrix correlation of CBZ, 3-OH CBZ, 10-OH CBZ and EP-CBZ in single and four solute systems, texture soils, organic matter and organic carbon.

Table S2. Results of the factorial analysis considering adsorption of studied compounds in the single-solute experiments and the physicochemical characteristics of the soils as variables and soils as cases.

Table.S3. Results of the factorial analysis considering adsorption of studied compounds in the four-solute experiments and the physicochemical characteristics of the soils as variables and soils as cases.

Table S4. Conditions applied in the batch experiments.

Table S5. LC-MS/MS parameters.

Table.S6. Instrumental limits of detection (LOD), instrumental limits of quantitation (LOQ) and intra- and interday precision, measured as relative standard deviation (n = 3), of the optimized methods.

Figure captains:

Figure S1. Evaluation of the equilibrium time on the sorption of CBZ, 3OH-CBZ, 10OH-CBZ and EP-CBZ onto the soil.

Figure S2. Evaluation of the soil/solution ratio on the sorption of CBZ, 3OH-CBZ, 10OH-CBZ and EP-CBZ onto the soil.

Figure S3. Evaluation of the influence of pH on the sorption of CBZ, 3OH-CBZ, 10OH-CBZ and EP-CBZ onto (A) soil 1 and (B) soil 3.

Figure S4. Linear, Freundlich and Langmuir models of CBZ adsorption on soil 1 in single-solute and four-solute systems.

Figure S5. Linear, Freundlich and Langmuir models of CBZ adsorption on soil 3 in single-solute and four-solute systems.

Figure S6. Linear, Freundlich and Langmuir models of 3OH-CBZ adsorption on soil 1 in single-solute and four-solute systems.

Figure S7. Linear, Freundlich and Langmuir models of 3OH-CBZ adsorption on soil 2 in single-solute and four-solute systems.

Figure S8. Linear, Freundlich and Langmuir models of 3OH-CBZ adsorption on soil 3 in single-solute and four-solute systems.

Figure S9. Linear, Freundlich and Langmuir models of 10OH-CBZ adsorption on soil 1 in the single-solute system.

Figure S10. Linear, Freundlich and Langmuir models of 10OH-CBZ adsorption on soil 3 in the single-solute system.

Figure S11. Linear, Freundlich and Langmuir models of EP-CBZ adsorption on soil 1 in single-solute and four-solute systems.

Figure S12. Linear, Freundlich and Langmuir models of EP-CBZ adsorption on soil 2 in single-solute and four-solute systems.

Figure S13. Linear, Freundlich and Langmuir models of EP-CBZ adsorption on soil 3 in single-solute and four-solute systems.

Table S1. Matrix correlation of CBZ, 3-OH CBZ, 10-OH CBZ and EP-CBZ in single and four solute systems, texture soils, organic matter and organic carbon.

	A_{CBZ} (s)	$A_{3OH\text{-}CBZ}$ (s)	$A_{10OH\text{-}CBZ}$ (s)	$A_{EP\text{-}CBZ}$ (s)	A_{CBZ} (m)	$A_{3\text{-}OHCBZ}$ (m)	$A_{EP\text{-}CBZ}$ (m)	Fine sand	Coarse sand	Silt	Clay	OM
A_{CBZ} (s)	**1.00**	**0.88**	**0.93**	**1.00**	0.60	0.02	0.32	**0.93**	-0.60	0.62	0.09	**0.79**
$A_{3OH\text{-}CBZ}$ (s)		**1.00**	**1.00**	**0.86**	**0.91**	0.48	**0.73**	**0.99**	**-0.90**	**0.92**	0.55	0.42
$A_{10OH\text{-}CBZ}$ (s)			**1.00**	**0.91**	**0.86**	0.39	0.66	**1.00**	**-0.86**	**0.87**	0.46	0.51
$A_{EP\text{-}CBZ}$ (s)				**1.00**	0.57	-0.03	0.28	**0.92**	-0.56	0.59	0.05	**0.82**
A_{CBZ} (m)					**1.00**	**0.81**	**0.95**	**0.85**	**-1.00**	**1.00**	**0.85**	0.00
$A_{3\text{-}OHCBZ}$ (m)						**1.00**	**0.95**	0.38	**-0.81**	**0.79**	**1.00**	-0.59
$A_{EP\text{-}CBZ}$ (m)							**1.00**	0.64	**-0.95**	**0.94**	**0.97**	-0.32
Fine sand								**1.00**	**-0.85**	**0.86**	0.45	0.52
Coarse sand									**1.00**	**-1.00**	**-0.86**	0.01
Silt										**1.00**	**0.84**	0.02
Clay											**1.00**	-0.53
OM												**1.00**

A_x: Adsorption of the compound *x*; OM: Organic matter

Table S2. Results of the factorial analysis considering adsorption of studied compounds in the single-solute experiments and the physicochemical characteristics of the soils as variables and soils as cases.

	Factor 1	Factor 2
A_{CBZ} (s)	0.57	**0.82**
$A_{3OH\text{-}CBZ}$ (s)	**0.89**	0.46
$A_{10OH\text{-}CBZ}$ (s)	**0.84**	0.54
$A_{EP\text{-}CBZ}$ (s)	0.54	**0.84**
Fine sand	**0.83**	0.56
Coarse sand	**-1.00**	-0.03
Silt	**1.00**	0.06
Clay	**0.87**	-0.50
MO	-0.04	**1.00**
Expl.Var	5.56	4.44
Prp.Totl	0.56	0.44

A_x: Adsorption of the compound *x*; OM: Organic matter

Table S3. Results of the factorial analysis considering adsorption of studied compounds in the four-solute experiments and the physicochemical characteristics of the soils as variables and soils as cases.

	Factor 1	Factor 2
A_{CBZ} (m)	**1.00**	0.04
$A_{3OH\text{-}CBZ}$ (m)	**0.83**	-0.56
$A_{EP\text{-}CBZ}$ (m)	**0.96**	-0.28
Fine sand	**0.83**	0.56
Coarse sand	**-1.00**	-0.03
Silt	**1.00**	0.07
Clay	**0.87**	-0.49
MO	-0.04	**1.00**
Expl.Var	6.05	2.95
Prp.Totl	0.67	0.33

A_x: Adsorption of the compound *x*; OM: Organic matter

Table S4. Conditions applied in the batch experiments

Equilibrium time optimization	
Agitation time	10, 20, 30, 40, 50, 60, 120, 720 and 1440 min
Shaking speed	40 rpm
Soil/solution ratio	2:10 w/v
Aqueous solution	9 mL of 0.01 M $CaCl_2$ solution
Temperature	25 ± 2 ºC
Concentration	1 mg L^{-1}
pH	Not controlled (measured pH 8)
Soil/solution ratio optimization	
Agitation time	24 h
Shaking speed	40 rpm
Soil/solution ratio	1:10, 2:10, 4:10 and 5:10 (w/v)
Aqueous solution	9 mL of 0.01 M $CaCl_2$ solution
Temperature	25 ± 2 ºC
Concentration	1 mg L^{-1}
pH	Not controlled (measured pH 8)
Influence of pH experiments	
Agitation time	24 h
Shaking speed	40 rpm
Soil/solution ratio	5:10 (w/v)
Aqueous solution	9 mL of 0.01 M $CaCl_2$ solution
Temperature	25 ± 2 ºC
Concentration	1 mg L^{-1}
pH	2, 4, 6, 8, 10 and 12
Adsorption isotherms on single- and four-solute systems	
Agitation time	24 h
Shaking speed	40 rpm
Soil/solution ratio	5:10 (w/v)
Aqueous solution	9 mL of 0.01 M $CaCl_2$ solution
Temperature	25 ± 2 ºC
Concentration	0.2, 0.3, 0.5, 0.8, 1 and 2 mg L^{-1}
pH	Not controlled (measured pH 8)

Table S5. LC-MS/MS parameters.

MS/MS parameters							
Compound	Ionization mode	Precursor ion (*m/z*)	Product ions (MRM1/MRM2) (*m/z*)	Fragmentor (V)	CE (V)	RT (min)	Ion ratio
CBZ	Positive	237.1	194.1/179.0	140	16/36	2.2	12.7
3OH-CBZ	Positive	253.1	210.1/167.0	120	16/40	2.4	23.3
10OH-CBZ	Positive	255.1	237.1/194.4	60	4/20	2.1	96.6
EP-CBZ	Positive	253.1	180.1/236.1	60	24/4	2.4	61.7

Table S6. Instrumental limits of detection (LOD), instrumental limits of quantitation (LOQ) and intra- and interday precision, measured as relative standard deviation (n = 3), of the optimized methods.

Compound	LOD (ng L^{-1})	LOQ (ng L^{-1})	Precision	
			Intraday (%)	Interday (%)
CBZ	1.5	5.0	1.8	8.0
3OH-CBZ	0.3	1.0	4.0	7.7
10OH-CBZ	0.3	1.0	3.0	8.8
EP-CBZ	0.1	0.5	9.0	11

Figure S1. Evaluation of the equilibrium time on the sorption of CBZ, 3OH-CBZ, 10OH-CBZ and EP-CBZ onto the soil.

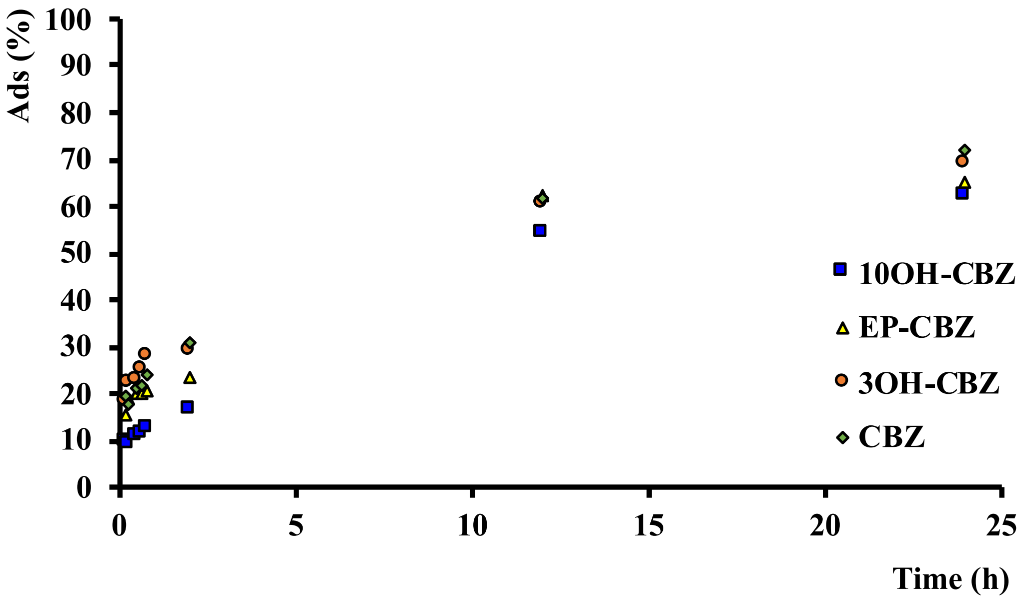

Figure S2. Evaluation of the soil/solution ratio on the sorption of CBZ, 3OH-CBZ, 10OH-CBZ and EP-CBZ onto the soil.

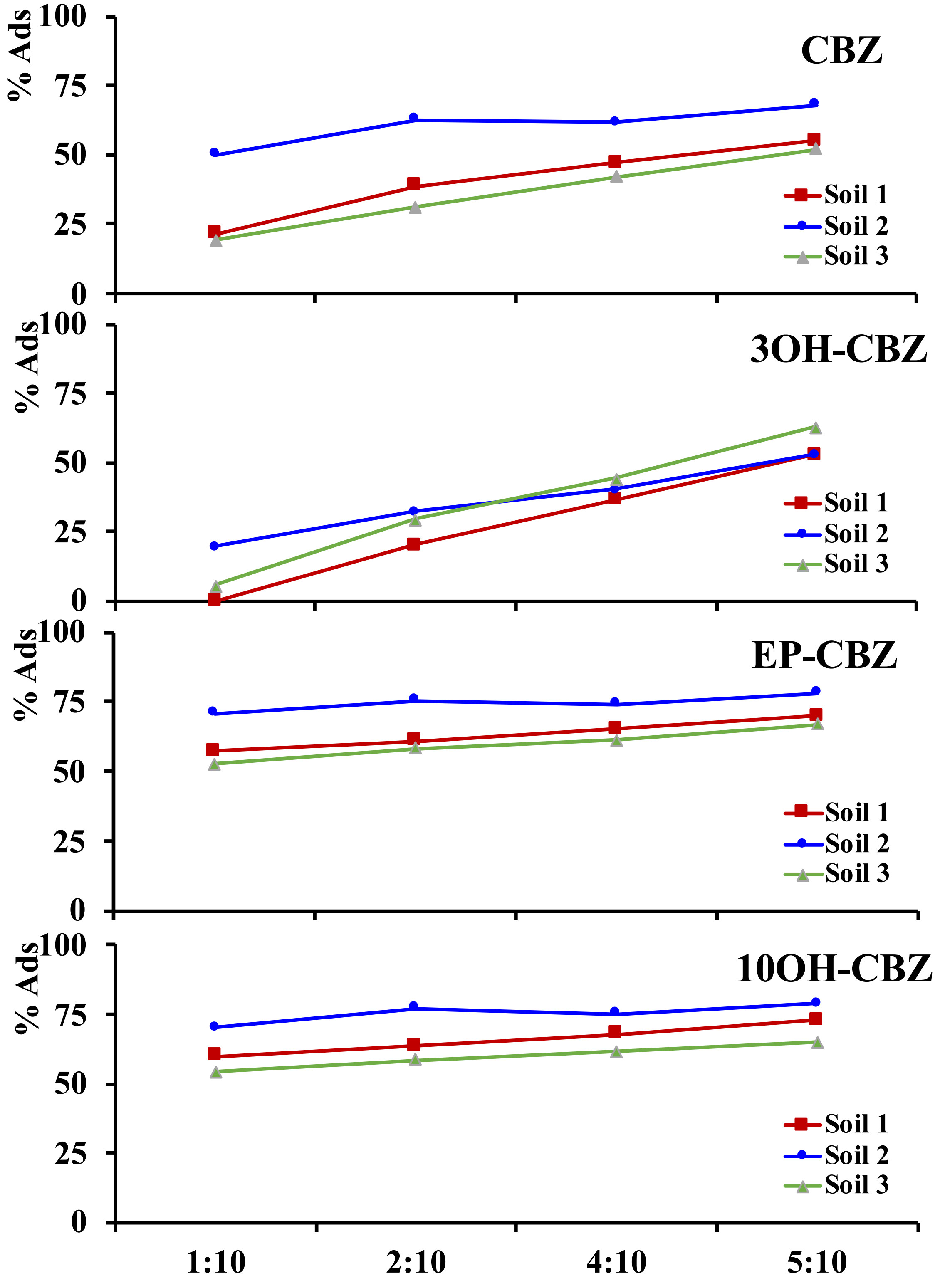

Figure S3. Evaluation of the influence of pH on the sorption of CBZ, 3OH-CBZ, 10OH-CBZ and EP-CBZ onto (A) soil 1 and (B) soil 3.

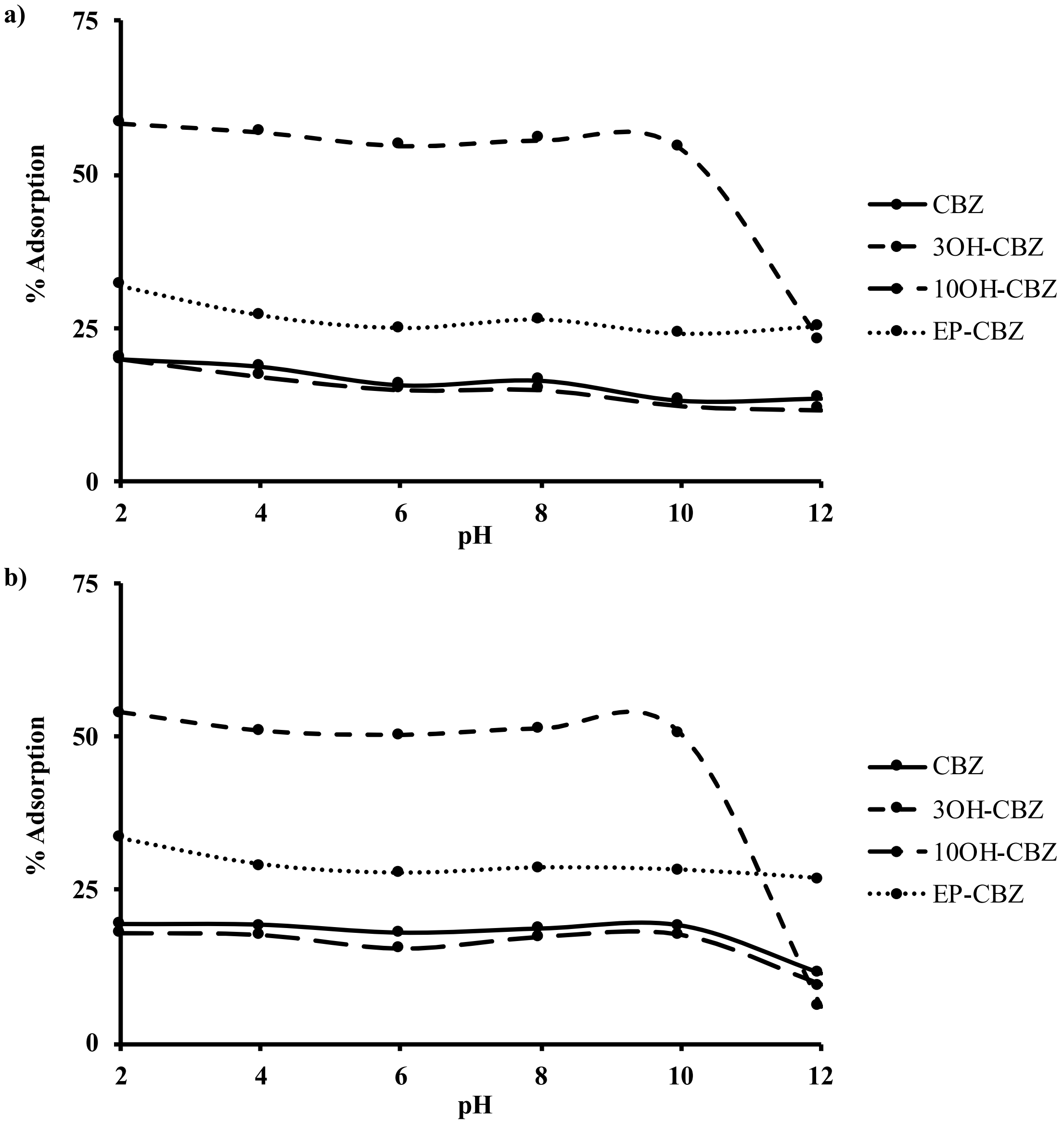

Figure S4. Linear, Freundlich and Langmuir models of CBZ adsorption on soil 1 in single-solute and four-solute systems.

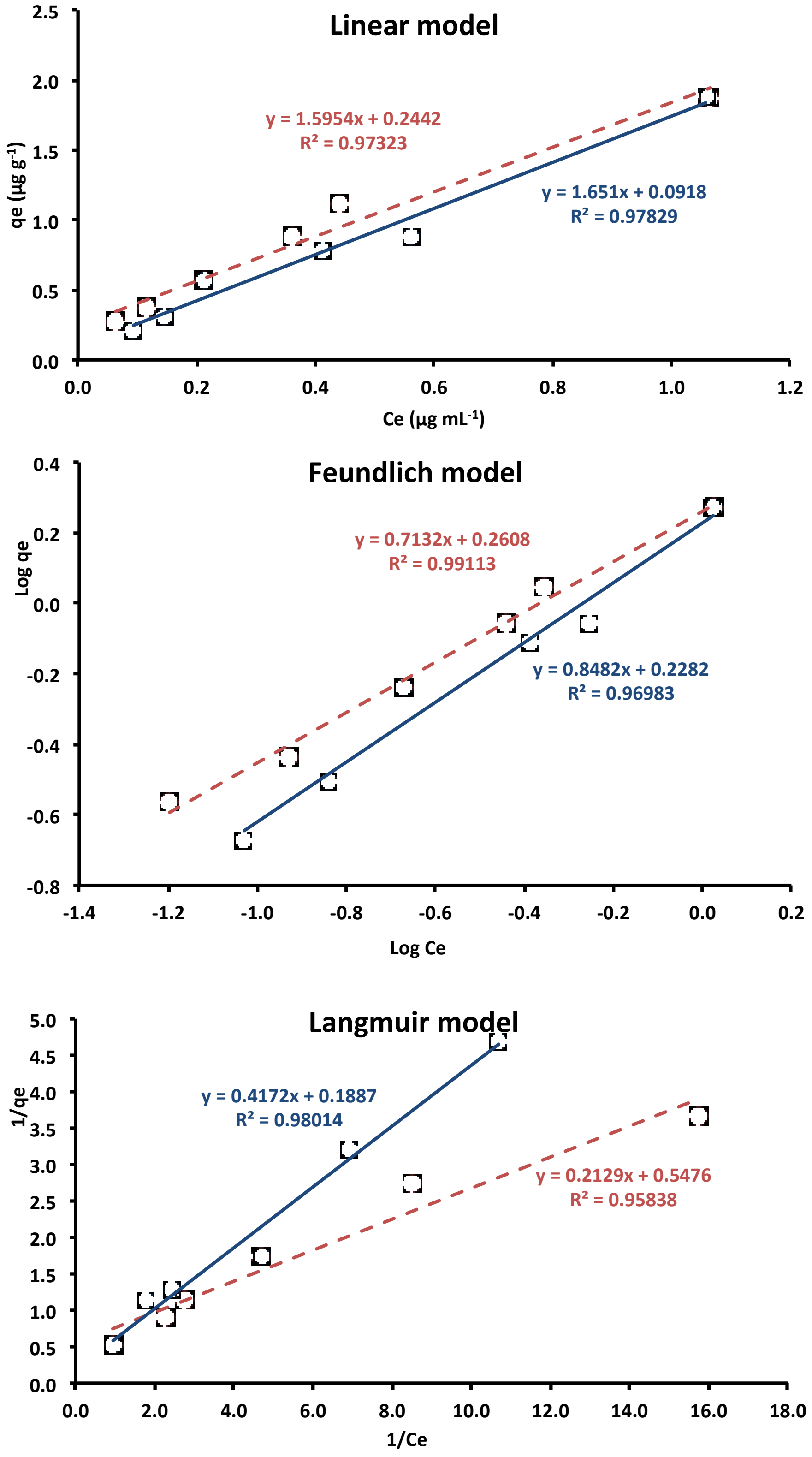

Figure S5. Linear, Freundlich and Langmuir models of CBZ adsorption on soil 3 in single-solute and four-solute systems.

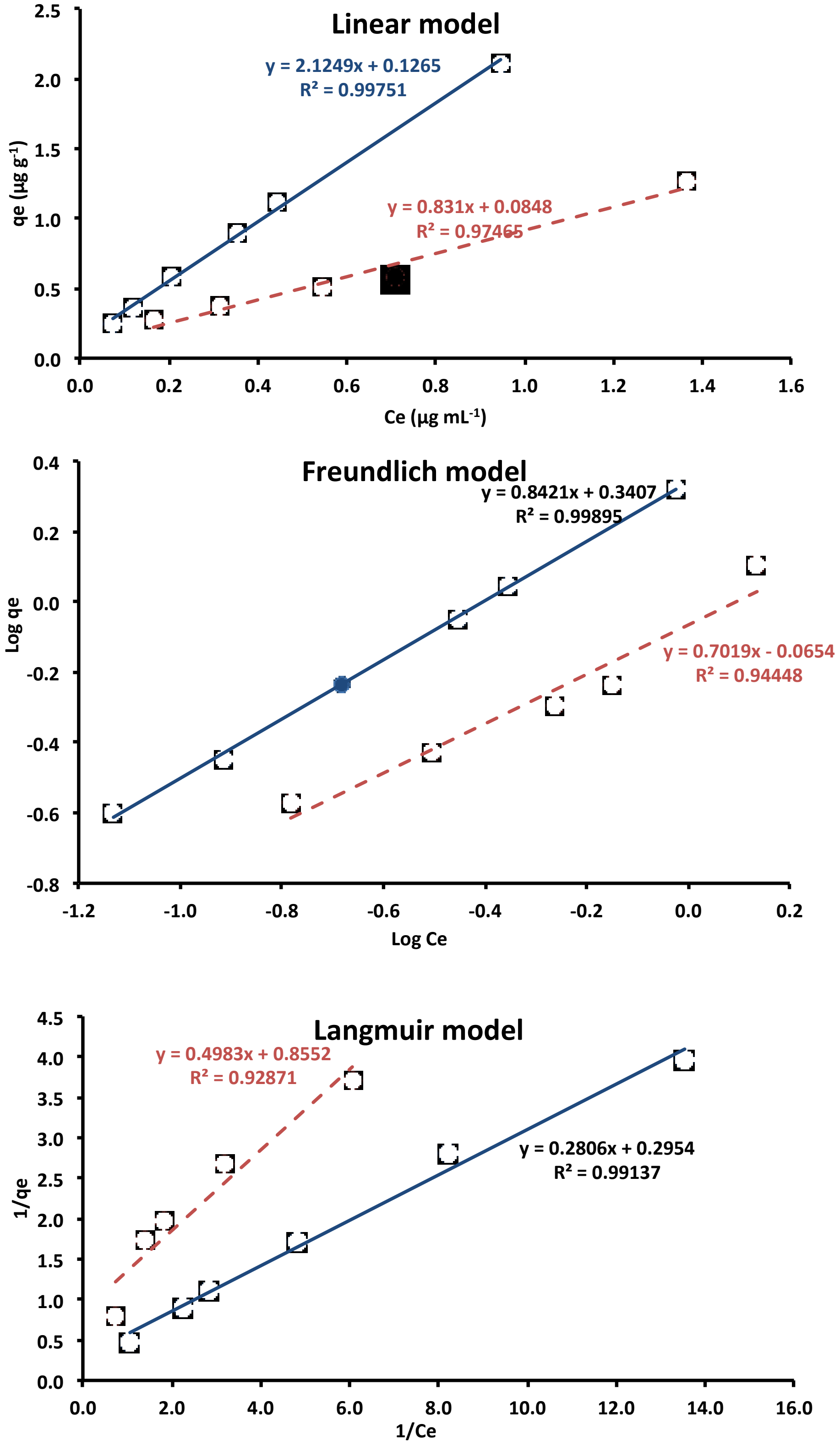

Figure S6. Linear, Freundlich and Langmuir models of 3OH-CBZ adsorption on soil 1 in single-solute and four-solute systems.

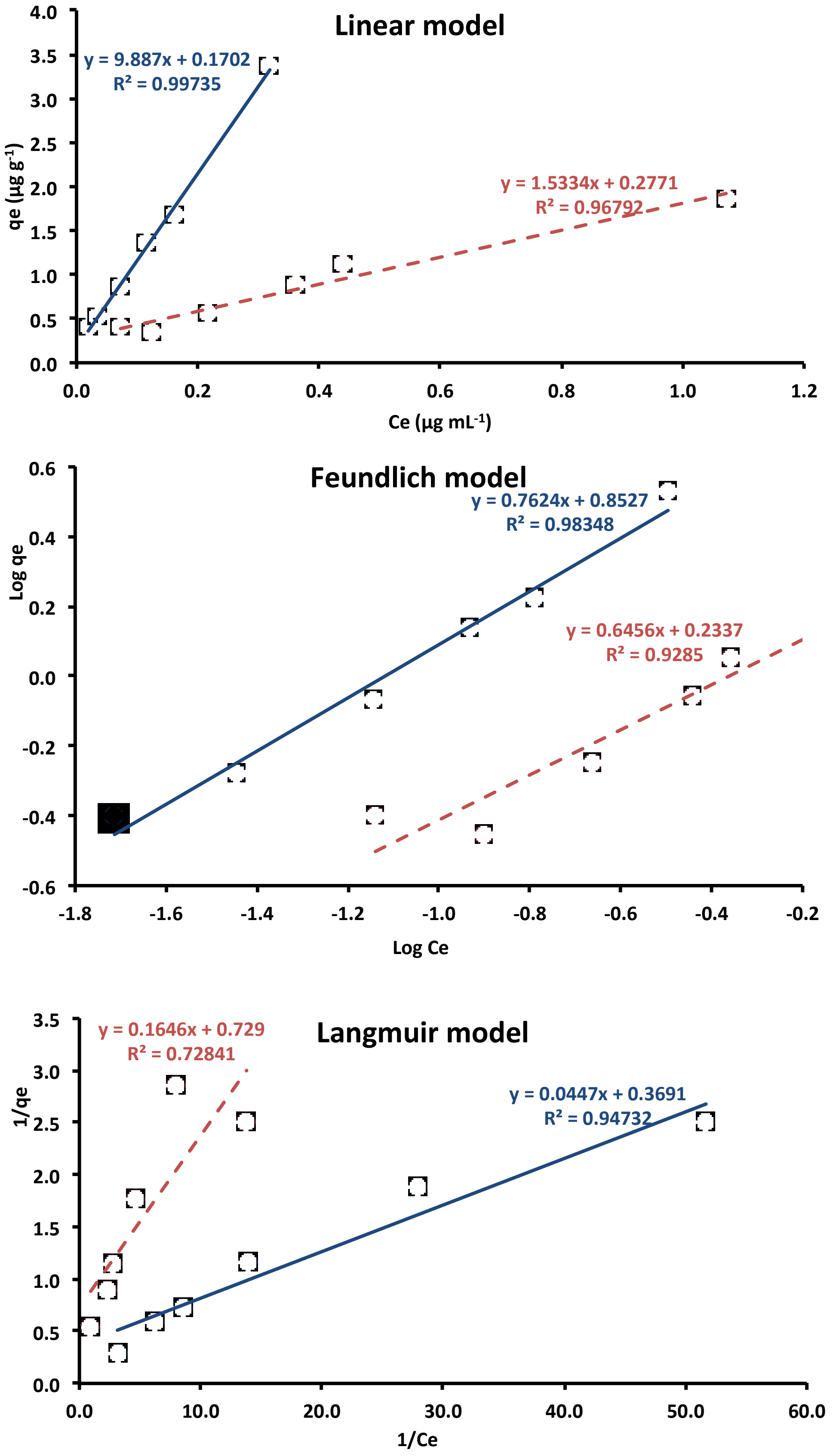

Figure S7. Linear, Freundlich and Langmuir models of 3OH-CBZ adsorption on soil 2 in single-solute and four-solute systems.

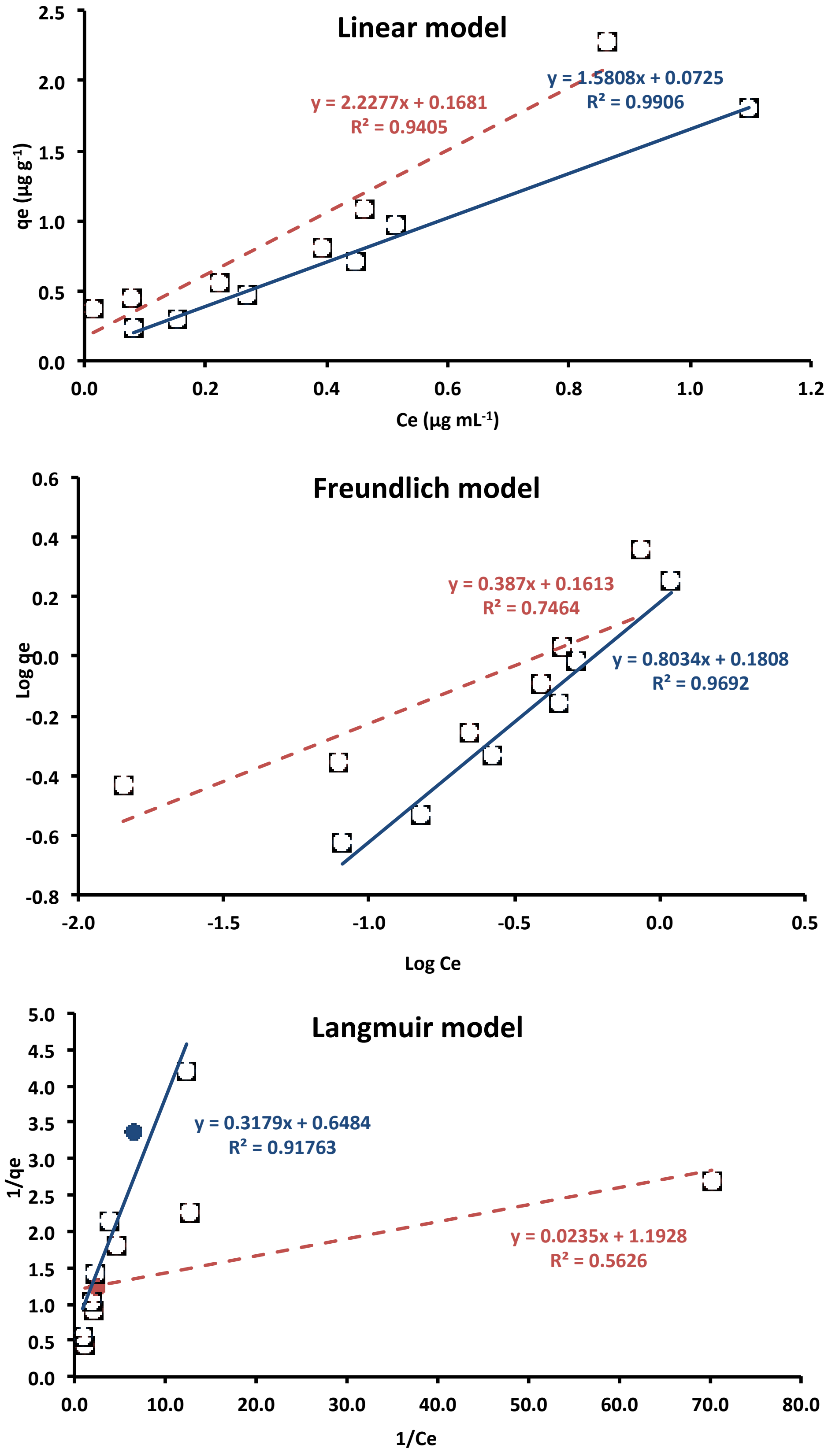

Figure S8. Linear, Freundlich and Langmuir models of 3OH-CBZ adsorption on soil 3 in single-solute and four-solute systems.

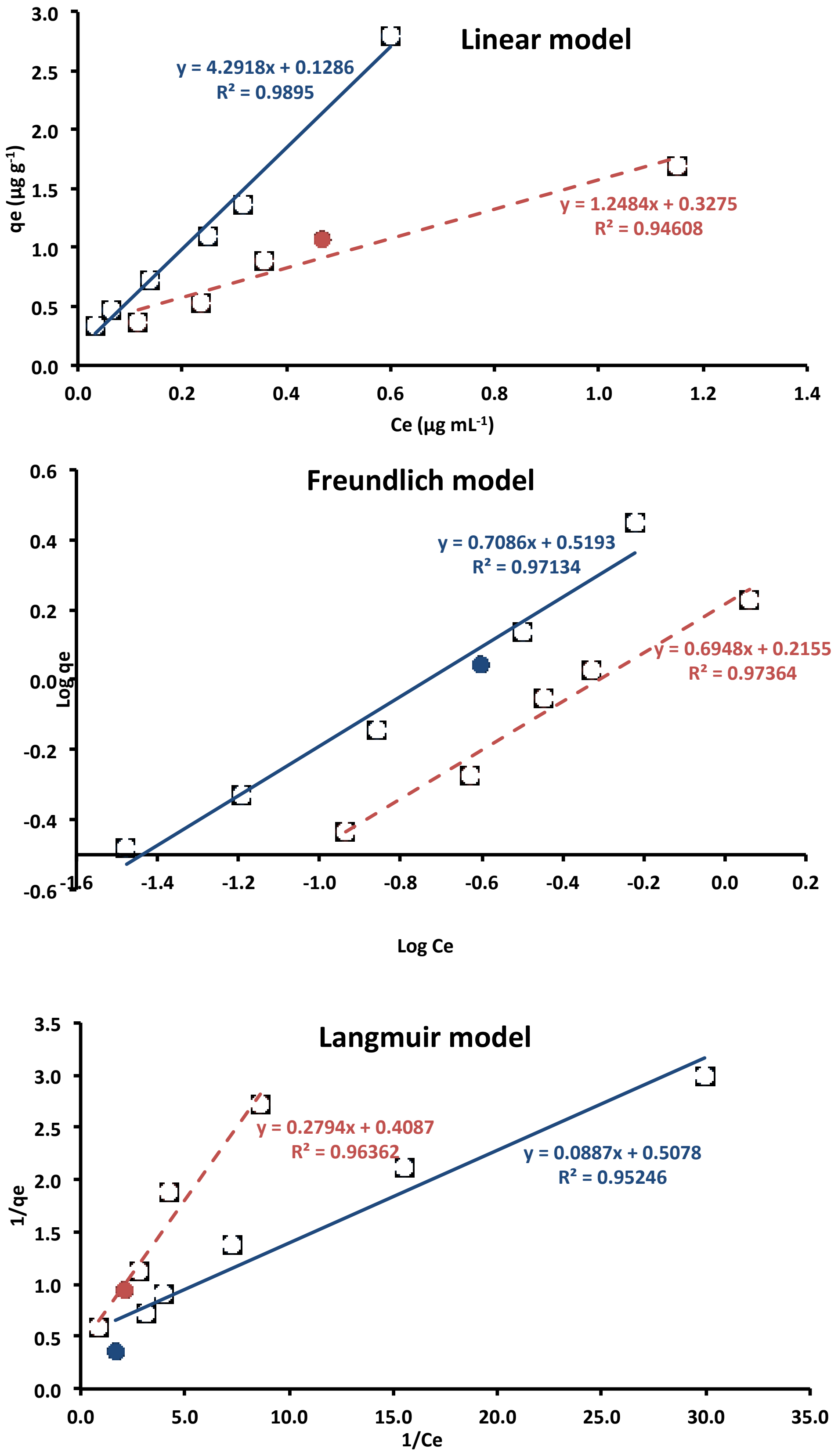

Figure S9. Linear, Freundlich and Langmuir models of 10OH-CBZ adsorption on soil 1 in the single-solute system.

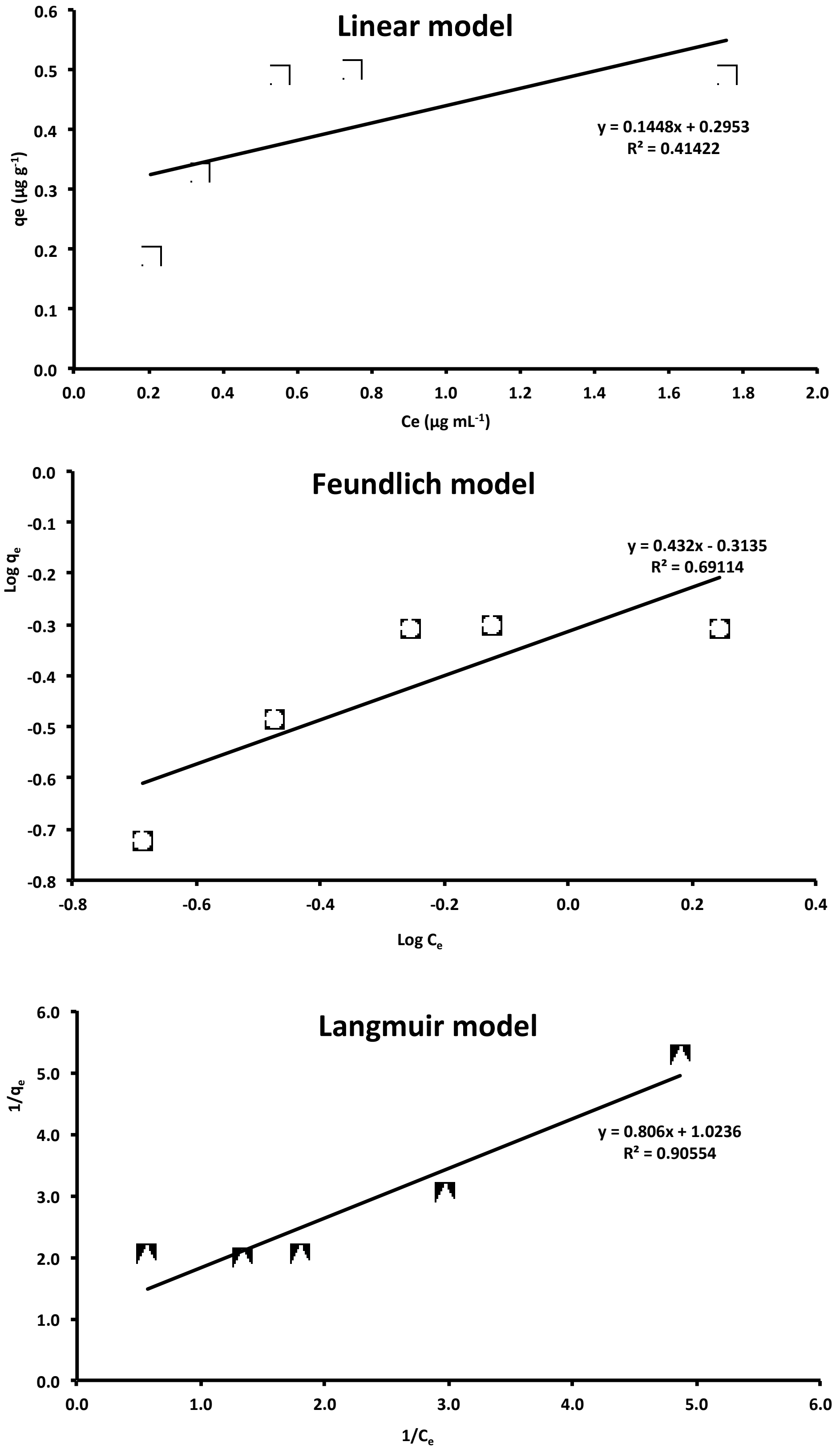

Figure S10. Linear, Freundlich and Langmuir models of 10OH-CBZ adsorption on soil 3 in the single-solute system.

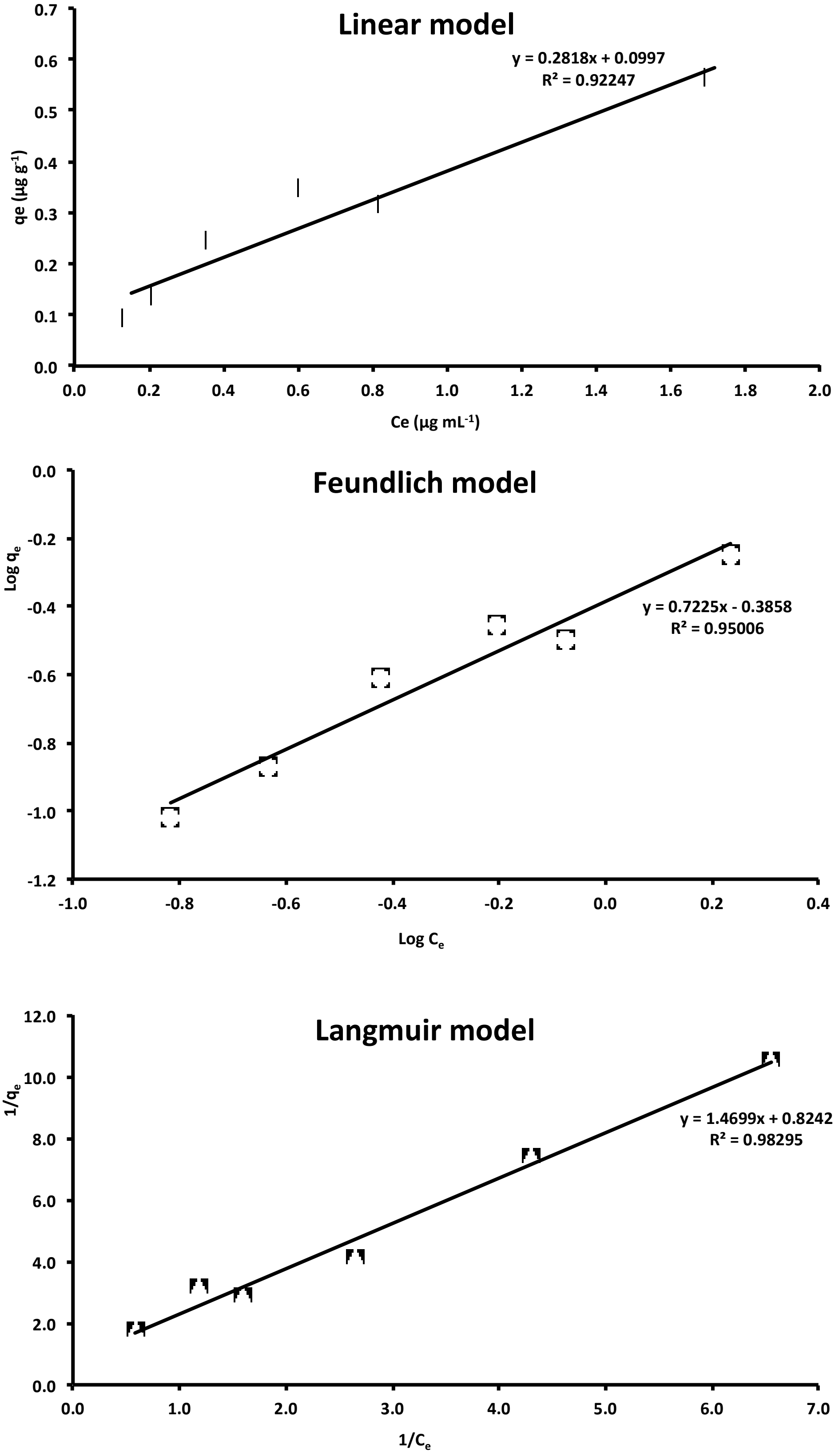

Figure S11. Linear, Freundlich and Langmuir models of EP-CBZ adsorption on soil 1 in single-solute and four-solute systems.

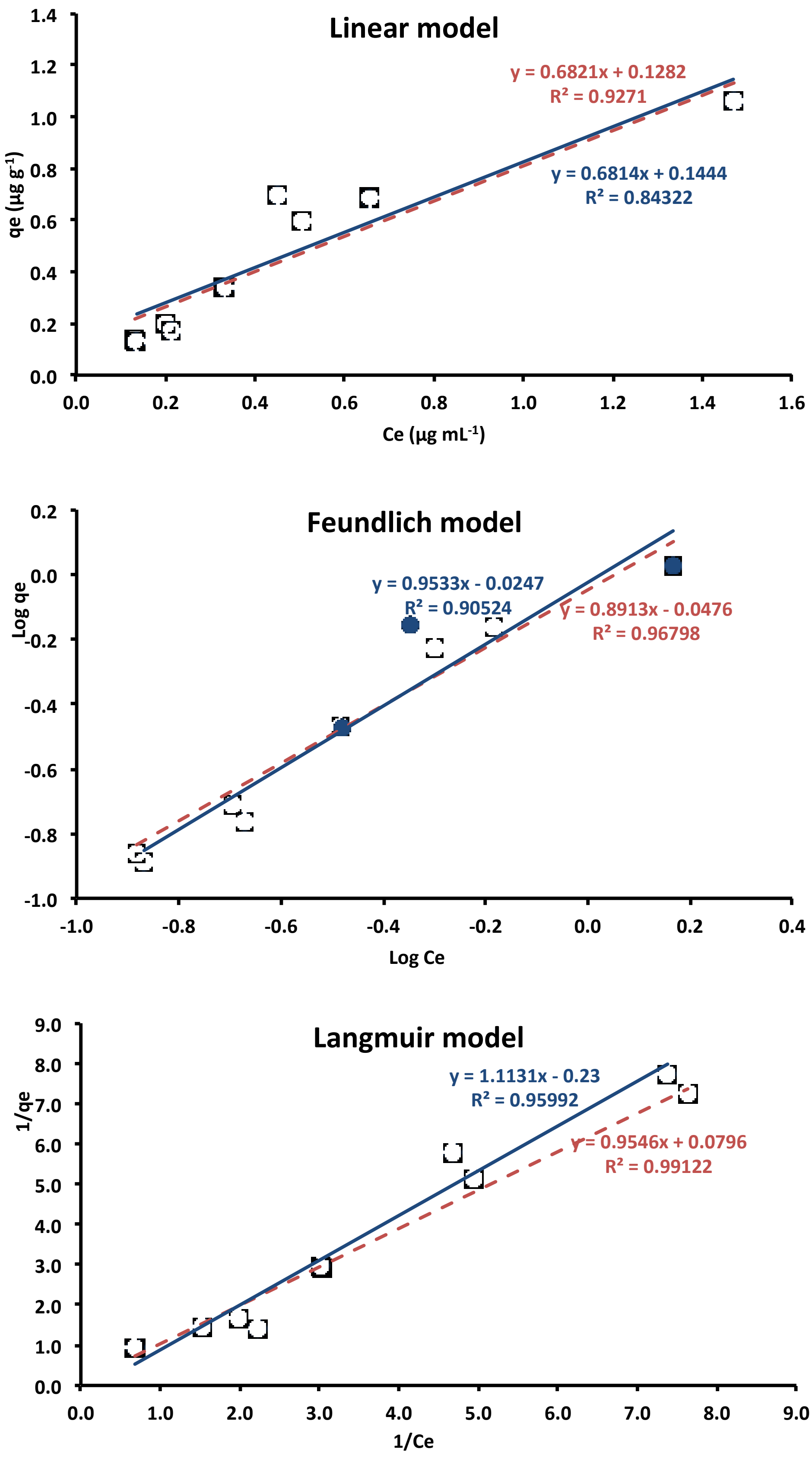

Figure S12. Linear, Freundlich and Langmuir models of EP-CBZ adsorption on soil 2 in single-solute and four-solute systems.

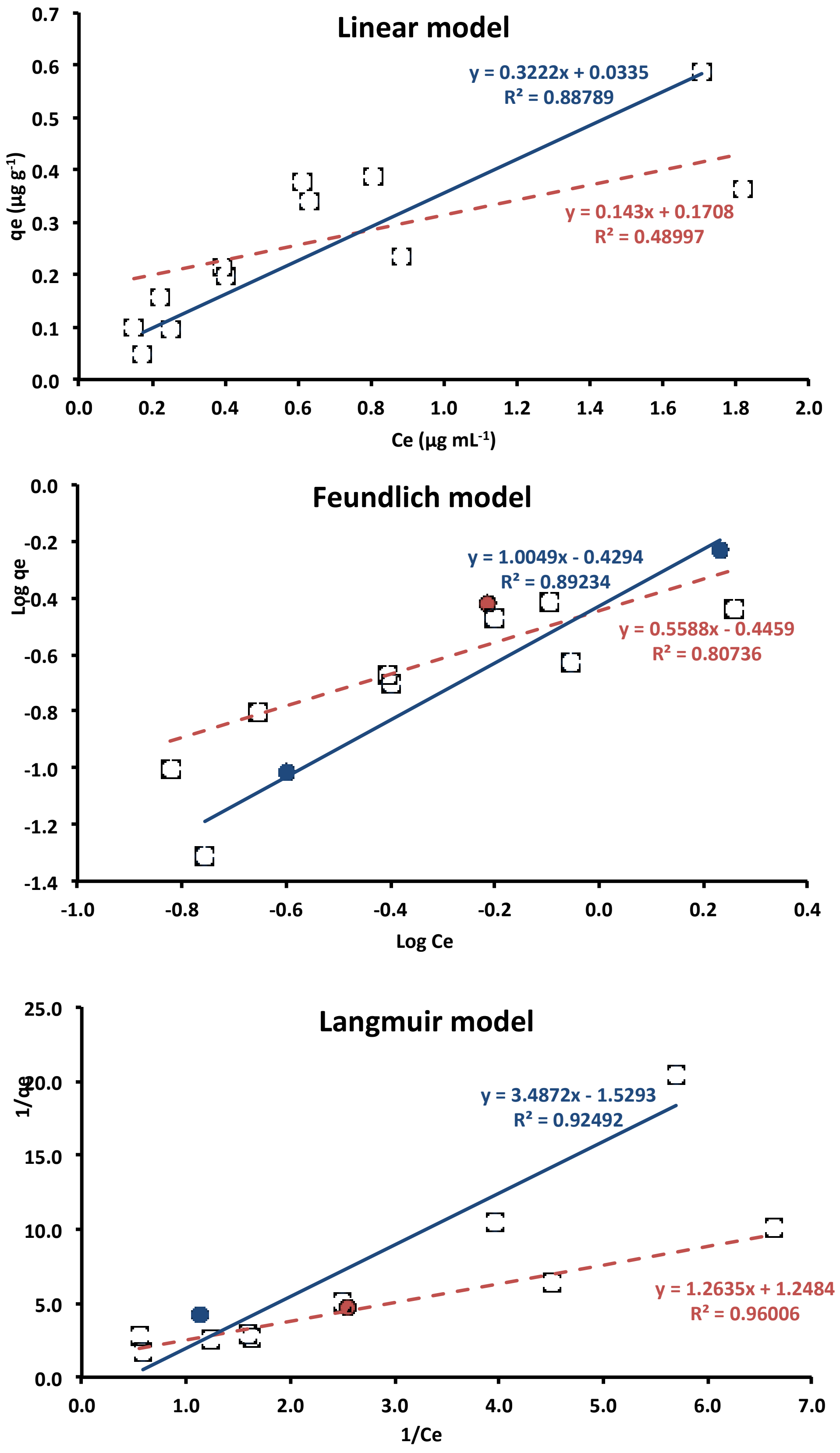

Figure S13. Linear, Freundlich and Langmuir models of EP-CBZ adsorption on soil 3 in single-solute and four-solute systems.

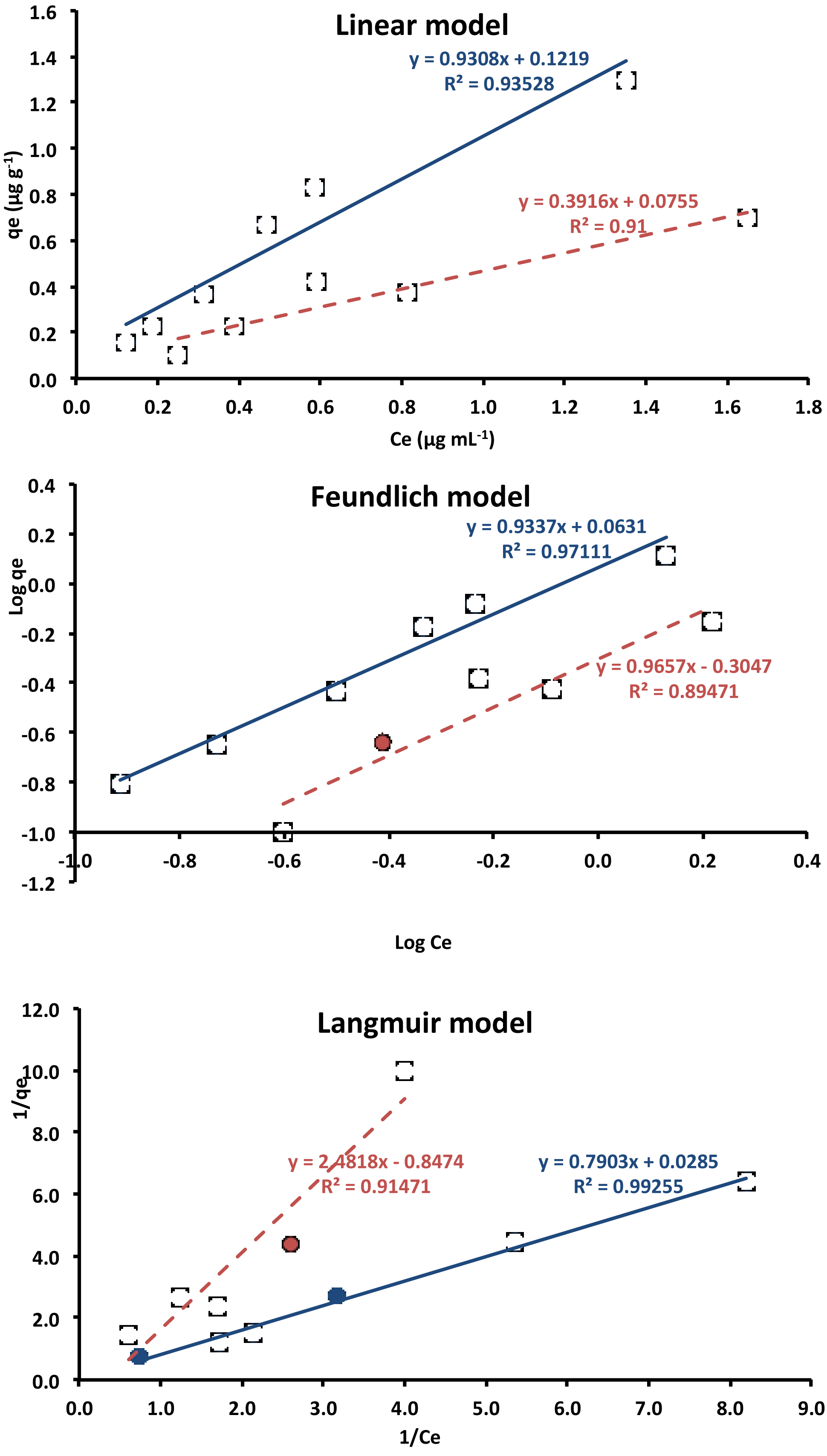

Supplement: Supplementary file 1 [file molecules-25-05306-s001.pdf]
